# Longitudinal markers of cerebral amyloid angiopathy and related inflammation in rTg-DI rats

**DOI:** 10.1038/s41598-024-59013-7

**Published:** 2024-04-10

**Authors:** Joseph M. Schrader, Feng Xu, Kevin J. Agostinucci, Nicholas A. DaSilva, William E. Van Nostrand

**Affiliations:** 1 Department of Biomedical and Pharmaceutical Sciences, George & Anne Ryan Institute for Neuroscience, University of Rhode Island, 130 Flagg Road, Kingston, Rhode Island 02881 USA; 2 Department of Molecular Biology, Cell Biology, and Biochemistry, Brown University, Providence, Rhode Island 02912 USA

**Keywords:** Cerebral amyloid angiopathy, Transgenic rats, Neuroinflammation, Proteomics, Biomarkers, Alzheimer's disease, Neuroscience, Diseases of the nervous system, Neuro-vascular interactions

## Abstract

Cerebral amyloid angiopathy (CAA) is a prevalent vascular dementia and common comorbidity of Alzheimer’s disease (AD). While it is known that vascular fibrillar amyloid β (Aβ) deposits leads to vascular deterioration and can drive parenchymal CAA related inflammation (CAA-ri), underlying mechanisms of CAA pathology remain poorly understood. Here, we conducted brain regional proteomic analysis of early and late disease stages in the rTg-DI CAA rat model to gain molecular insight to mechanisms of CAA/CAA-ri progression and identify potential brain protein markers of CAA/CAA-ri. Longitudinal brain regional proteomic analysis revealed increased differentially expressed proteins (DEP) including ANXA3, HTRA1, APOE, CST3, and CLU, shared between the cortex, hippocampus, and thalamus, at both stages of disease in rTg-DI rats. Subsequent pathway analysis indicated pathway enrichment and predicted activation of TGF-β1, which was confirmed by immunolabeling and ELISA. Further, we identified numerous CAA related DEPs associate with astrocytes (HSPB1 and MLC1) and microglia (ANXA3, SPARC, TGF-β1) not previously associated with astrocytes or microglia in other AD models, possibly indicating that they are specific to CAA-ri. Thus, the data presented here identify several potential brain protein biomarkers of CAA/CAA-ri while providing novel molecular and mechanistic insight to mechanisms of CAA and CAA-ri pathological progression and glial cell mediated responses.

## Introduction

Cerebral amyloid angiopathy (CAA) is a prevalent cerebral small vessel disease (CSVD), prominent contributor to vascular cognitive impairment and dementia (VCID) in the elderly, and the most common vascular comorbidity in Alzheimer’s disease (AD)^1–3^. Arising from deposition of amyloid β (Aβ) fibrils in the cerebral vasculature, CAA can cause cerebral vessel wall dysfunction, cerebral infarction, intracerebral hemorrhages (ICH) and microbleeds, and white matter (WM) damage which contribute to cognitive dysfunction^1,4,5^. CAA can present as two distinct types, CAA type-1 where Aβ deposition occurs primarily in cortical capillaries and microvessels with “dyshorric” amyloid protruding into the adjacent parenchyma, and CAA type-2 where Aβ deposition occurs in larger vessels, mainly present in the meningeal and intracortical small arteries and arterioles^1,6,7^. Dyshorric amyloid in CAA type-1 results in severe and chronic perivascular neuroinflammation (CAA-ri) and neurodegeneration ultimately correlating with cognitive decline and dementia^6–9^. Currently, clinical CAA diagnosis is based on prominent neuroimaging characteristics including macrohemorrhages, microbleeds, superficial siderosis, and white matter hyperintensities, and thus, only possible in advanced disease stages, with definitive diagnosis often only occurring post-mortem^3,5^. Despite the prevalent clinical burden, the underlying mechanisms of CAA remain poorly understood, and there exists no approved treatment, nor validated diagnostic biomarkers to detect CAA in general nor to distinguish between CAA type-2 and CAA type-1 with prominent CAA-ri.

Proteomic analysis of preclinical animal models utilizing protein mass spectrometry has become a popular approach for biomarker discovery in a variety of diseases ^10–13^. Data-independent acquisition (DIA) approaches followed by peptide library referencing can deliver quantitative analysis of thousands of proteins within a single sample^14,15^, and are an exceptional tool for proteomic analysis and the identification of potential disease biomarkers in preclinical animal models.

Previously, we developed a preclinical rat model of CAA type-1, rTg-DI, that produces Dutch (E22Q)/Iowa (D23N) familial CAA mutant Aβ in the brain^16^. The rTg-DI model faithfully recapitulates many aspects of human CAA type-1 pathologies including robust CAA-ri, cerebral microhemorrhages, capillary pericyte degeneration, microvessel thrombotic occlusions, axonal damage, WM degeneration, and cognitive deficits^16–19^. Despite early-onset and progressive microvascular amyloid deposition, rTg-DI rats fail to develop appreciable larger vessel CAA-type 2 or parenchymal fibrillar Aβ plaques^16,17^. Therefore, the rTg-DI rat is a well characterized and faithful preclinical model of CAA type-1 and CAA-ri.

Earlier, we conducted brain regional proteomic analysis of rTg-DI rats with late stage disease (12 M) and identified numerous differentially expressed proteins (DEPs) in the cortex, hippocampus, thalamus, and corpus callosum regions^20,21^. More recently, we performed comparative proteomic analysis of the rTg-DI rat brain with the spontaneously hypertensive stroke-prone (SHR-SP) rats ^22^, a well characterized non-amyloidal CSVD model of chronic hypertension^23–25^, and identified DEPs distinguishing the rTg-DI CAA model from the SHR-SP hypertensive model. At 12 M the rTg-DI rats exhibits extensive capillary amyloid deposition, strong CAA-ri, and numerous vessel occlusions and microbleeds, and therefore represent late stages of CAA when neuroimaging characteristics are apparent^16,18,20,21^. Since useful diagnosis requires biomarkers detectable in early disease stages, the potential of previously identified rTg-DI DEPs as biomarkers requires further investigation in earlier disease stages.

Here we present regional proteomic analysis of rTg-DI rat brains in emergent disease stages for the identification of DEPs. Additionally, raw spectral files from 12 M rTg-DI rat brain regions generated in our previous study^20^, were re-analyzed here in parallel, for a comparison of longitudinal DEPs, particularly associated with CAA-ri. Subsequent pathway analysis provided mechanistic insight to the early progression of CAA-ri related pathology. Thus, we describe several potential biomarkers of CAA-ri, with early and sustained expression in the rTg-DI rat model of CAA and report potential molecular mechanisms of initial pathological progression.

## Results

### rTg-DI rats exhibit progressive microvascular amyloid accumulation

**Figure 1:**
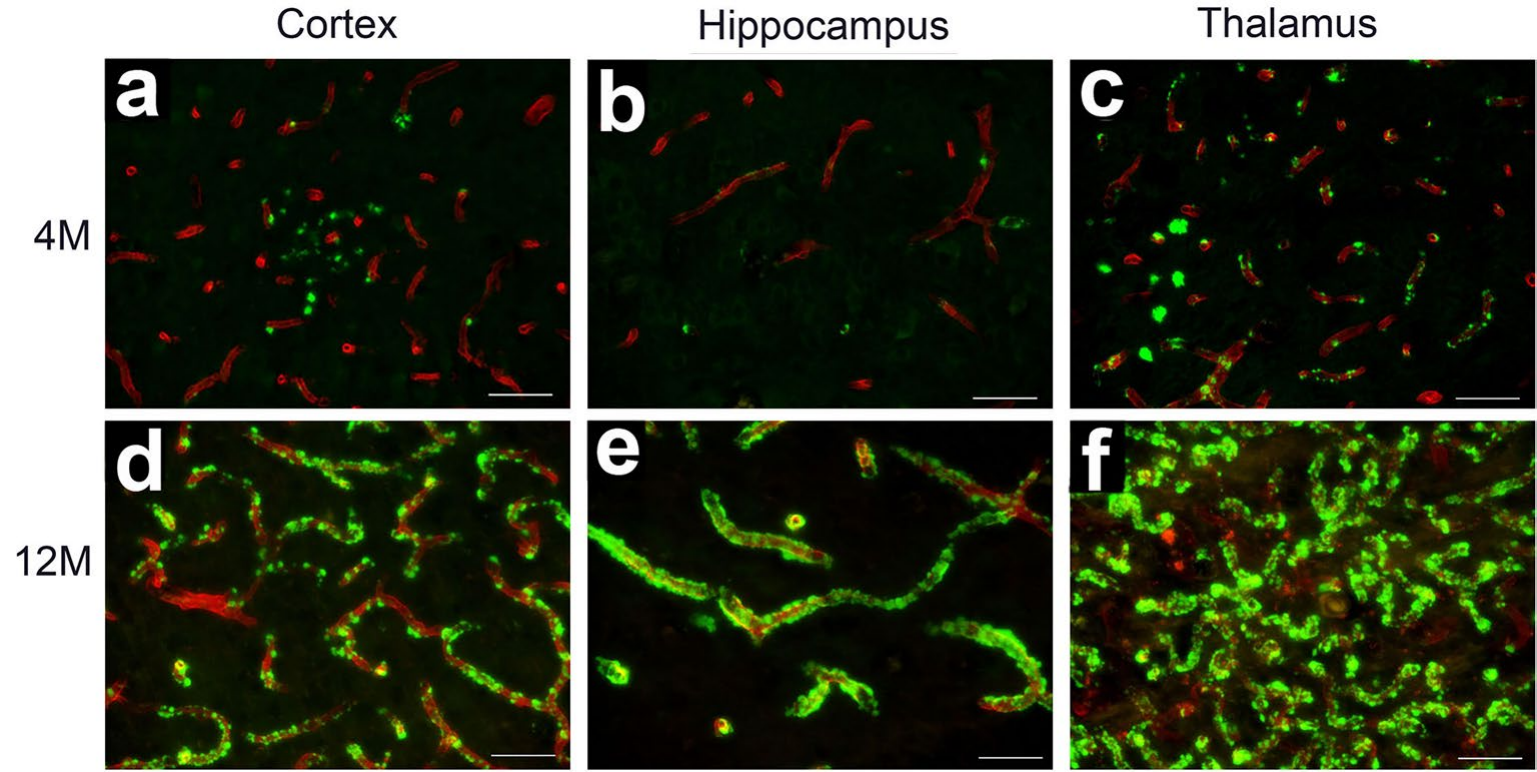
Cerebral microvascular amyloid in 4 M and 12 M rTg-DI rat brain. Brain sections from 4 M (**a**,**b**,**c**) and 12 M (**d**,**e**,**f**) cortex, hippocampus and thalamus, respectively, of rTg-DI rats were immunolabeled with rabbit polyclonal antibody to collagen IV to specifically detect cerebral microvessels (red) and stained with thioflavin S to identify fibrillar amyloid (green). Emerging cerebral microvascular fibrillar amyloid deposits were observed in 4 M rTg-DI rats, while prominent fibrillar amyloid deposits were observed in 12 M rTg-DI rats. Scale bars = 50 µm.

We previously demonstrated early onset and age-dependent accumulation of microvascular amyloid in several brain regions of the rTg-DI rat model, beginning at ≈3 M^16,17,20,21^. At each stage of disease, the presence of microvascular CAA occurs in the thalamus > hippocampus > cortex, although at 12 M all regions display moderate/severe microvascular Aβ accumulation^16,17,20^. Figure 1 presents representative images displaying early stages of microvascular Aβ deposition in the cortex, hippocampus, and thalamus at 4 M (Fig. 1a–c), and dramatically increasing at 12 M (Fig. 1e–f) demonstrating the present cohort of rTg-DI rats is consistent with the previous reported timeline of pathology^16,17,20^.

### Increase activated glial cells reveals CAA-ri in emergent disease in rTg-DI rats

**Figure 2:**
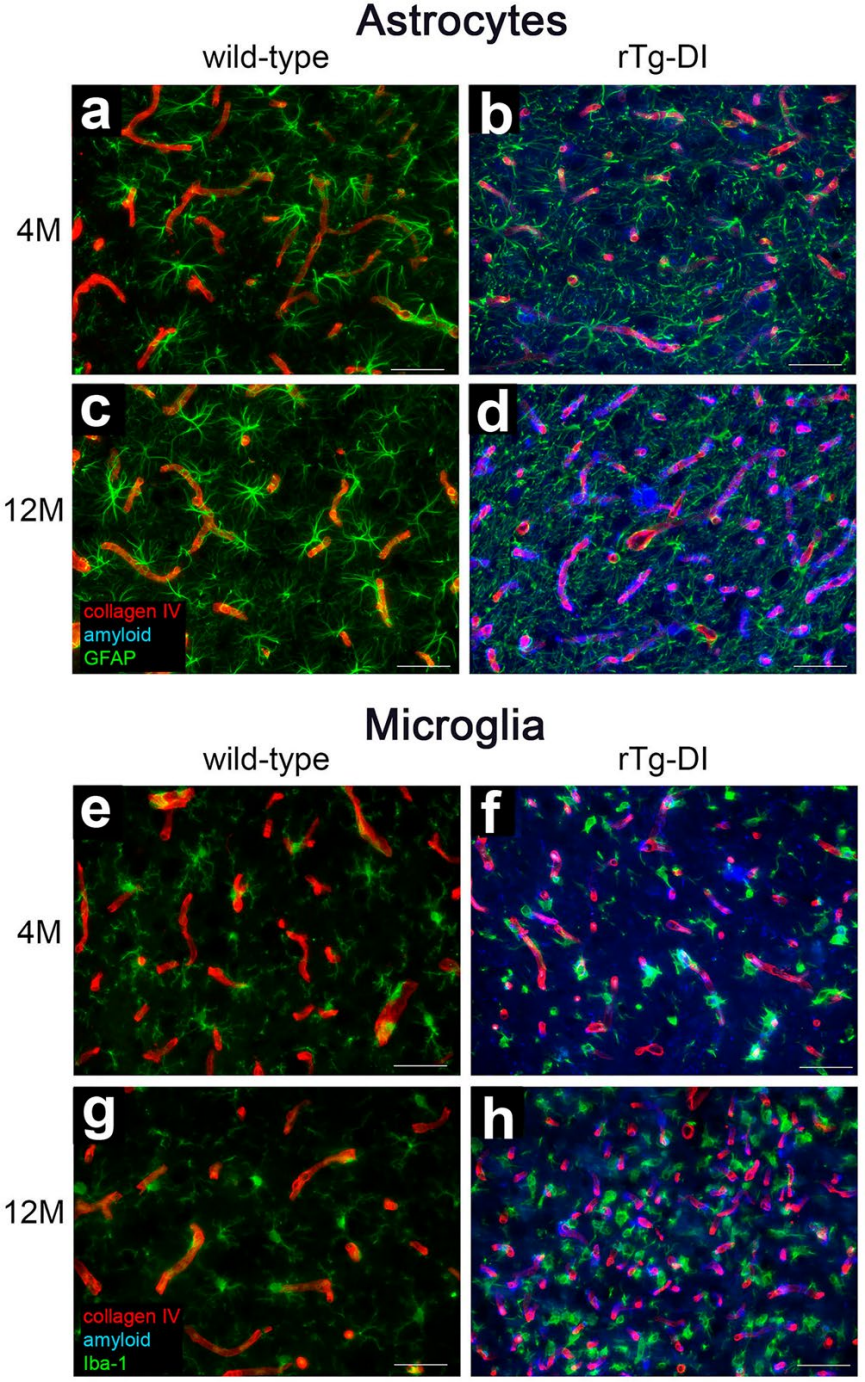
Increased astrocytes and microglia in 4 M and 12 M rTg-DI rats. Brain sections from 4 M WT (**a**,**e**) and rTg-DI rats (**b**,**f**) and 12 M WT (**c**,**g**) and rTg-DI rats (**d**,**h**) were labeled with Amylo-Glo to detect microvascular fibrillar amyloid (blue), goat polyclonal antibody to collagen IV to detect cerebral microvessels (red), and rabbit polyclonal antibody to GFAP to detect astrocytes (**a**–**d**) (green) or rabbit polyclonal antibody to Iba-1 to detect microglia (e–h) (green). Images from the thalamus are shown. Scale bars = 50 µm.

Cerebral microvascular amyloid deposition in rTg-DI rats induces severe CAA-ri and marked enhancement of activated glial cells^16,17,20^. At disease onset, astrocyte densities are increased in all brain regions in rTg-DI rats compared with WT rats, whereas at 12 M with advanced stages of disease, astrocyte densities are further increased > 2.5-fold in all brain regions^17,20^. Similarly, age-dependent elevation in activated microglia in the rTg-DI rats occurs as well, beginning at the onset of microvascular CAA deposition and further increasing at 12 M compared with WT rats^17,20^. Corresponding with CAA levels, enhancement of glial cells is most dramatic in the thalamus. Figure 2 shows representative images from the thalamus of 4 M (Fig. 2b,f) and 12 M rTg-DI (Fig. 2d,h) rats displaying increased activated astrocytes and microglia in this present cohort is consistent with our previous findings^17,20^. Although early-stage CAA-ri and glial activation are present in the 4 M cohort we observed no evidence of microbleeds indicating that this key late-stage pathology has not yet developed at this emergent stage of disease (data not shown).

### Differentially expressed proteins in rTg-DI rat brain regions

**Figure 3:**
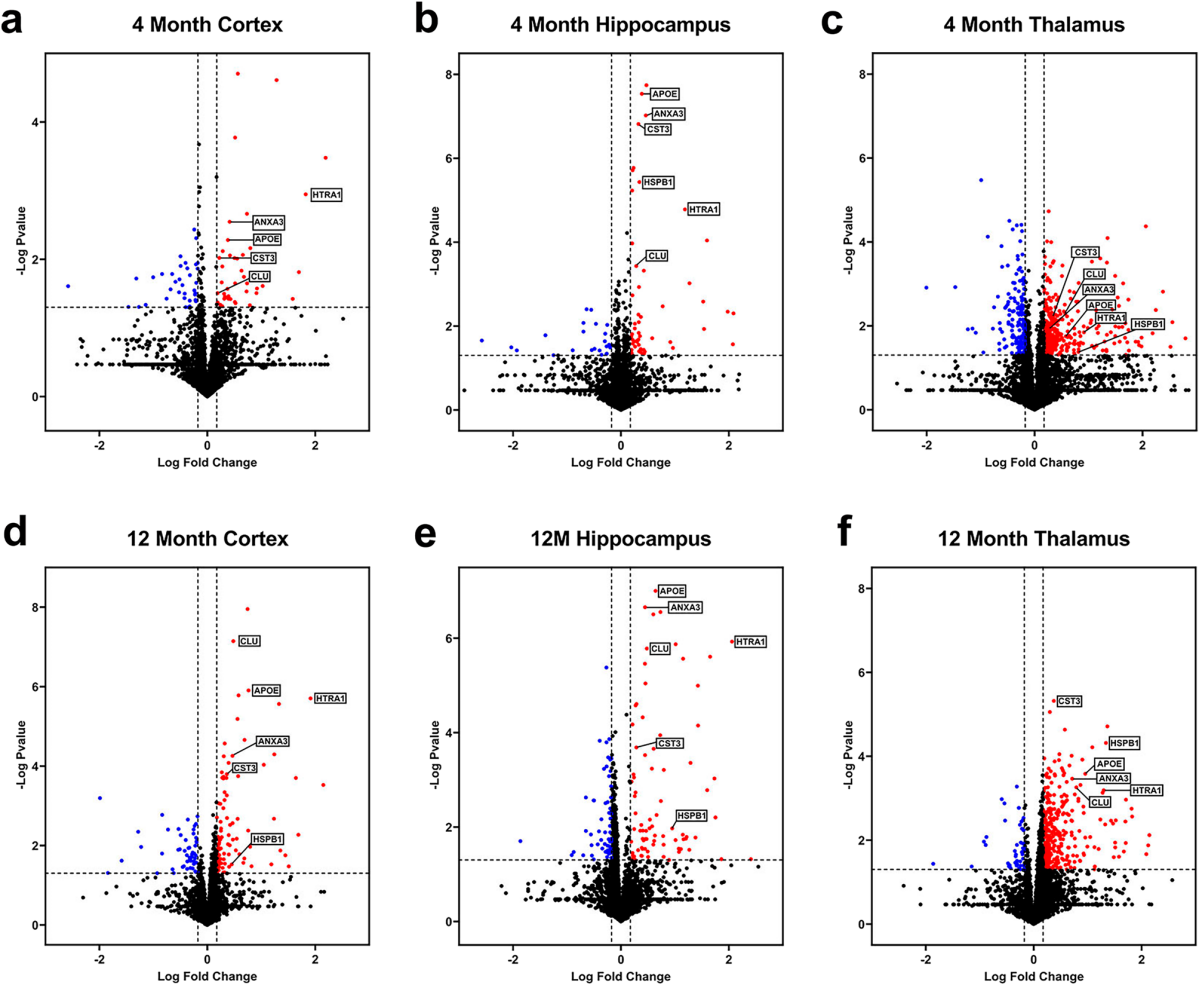
Differentially expressed proteins (DEPs) in 4 M and 12 M rTg-DI rat brain regions. Volcano plots generated from Log Fold Change and -Log *p* values for all proteins analyzed in 4 M (**a**–**c**) and 12 M (**d**–**f**) cortex, hippocampus, and thalamus, respectively, from rTg-DI rat brains. Points representing upregulated proteins (≥ 50% increase, *p* ≤ 0.05) and downregulated (≥ 33% decrease, *p* ≤ 0.05) proteins relative to WT rats are shown in red and blue, respectively.

The above findings show onset of amyloid deposition and enhancement of astrocytes and microglia, indicating that at 4 M rTg-DI rats represent an emergent stage of CAA-type 1 with developing CAA-ri, that will progress with more extensive vascular amyloid, CAA-ri, and vasculopathies at 12 M. Consequently, rTg-DI rats at 4 M may harbor an altered proteomic signature including biomarker candidates predictive of pathological progression evident at 12 M. Therefore, we compared cortical, hippocampal, and thalamic regional proteomic changes of 4 M and 12 M rTg-DI rats. Raw spectral files from our previous brain regional analysis of 12 M rTg-DI rats, which are publicly available through the MassIVE Repository^20^, were reanalyzed in parallel for comparative analysis. Though the raw spectral files from the 12 M rTg-DI rats have been reported earlier, the analysis performed here included an updated spectral library in combination with the Biognosys rat brain, liver, and kidney standard library. Additionally, the present analysis included the 4 M rTg-DI regional samples and was performed with an updated Spectronaut version (Spectronaut 17.5.230413.55965) utilizing the “local” normalization strategy for quantification rather than “global”. Thus, the analysis described here presents additional protein identifications, and novel protein quantification data compared with our previously published study^20^. While some differences in the results between our past and current analysis were observed in proteins of minor interest, differential expression of proteins highlighted below were largely repeated between the analyses. A total of nearly 4000 proteins were identified and quantified by Spectronaut, and the number of proteins identified and quantified in each analyzed sample is displayed in Supplemental Figure S1. Differentially expressed proteins were defined as proteins with either ≥ 50% increase or ≥ 33% decrease compared with age matched WT expression, and *p* < 0.05 as described in the “Methods” section. Volcano plots of all analyzed proteins in each region from each age group are depicted in Fig. 3, with DEPs colored in red or blue for significantly increased or decreased, respectively, and markers of specific interest are labeled. 42, 54, and 343 significantly elevated proteins were identified in the cortex, hippocampus, and thalamus of 4 M rTg-DI rats, respectively, while 37, 23, and 165 proteins were found significantly reduced in the cortex, hippocampus, and thalamus, respectively (Table S1-S3). Meanwhile, 92, 81, and 350 proteins were found significantly elevated in the 12 M rTg-DI cortex, hippocampus, and thalamus, respectively, while 52, 59, and 43 proteins were significantly reduced in the cortex, hippocampus, and thalamus, respectively (Table S4–S6).

**Figure 4:**
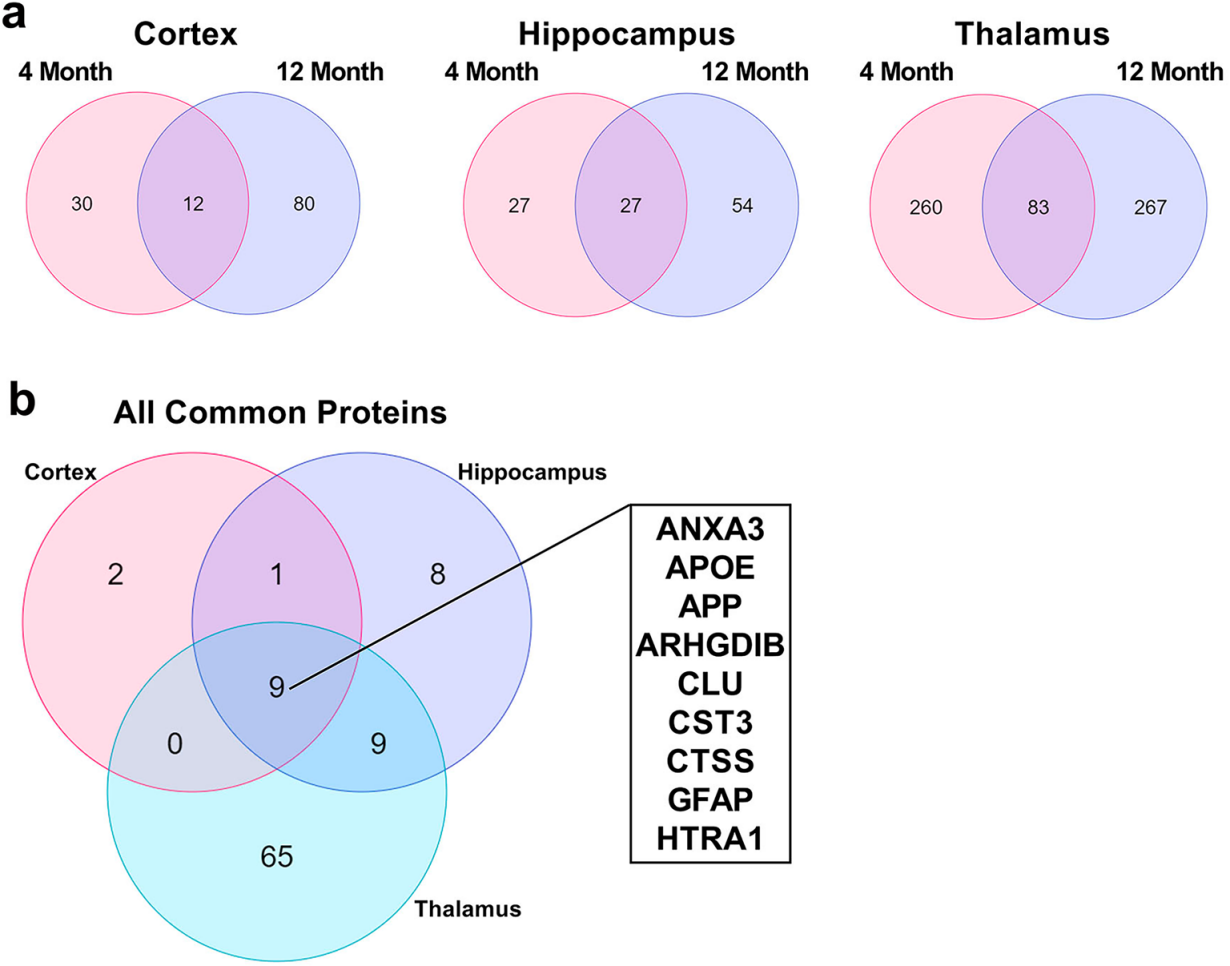
Upregulated protein comparison in 4 M and 12 M rTg-DI brains. (**a**) Venn diagrams comparing significantly enhanced proteins (≥ 50% increase, *p* ≤ 0.05, n = 6) occurring at both 4 M and 12 M in the rTg-DI cortex, hippocampus, and thalamus. (**b**) Venn diagram comparing proteins elevated at both 4 M and 12 M in the cortex, hippocampus, and thalamus, including a list of proteins elevated at both 4 M and 12 M in all three regions.

To identify longitudinal DEPs, we compared the elevated proteins in each reach region in each age group, and Venn diagrams of the DEPs comparisons are displayed in Fig. 4a. Here 122 proteins were elevated within at least one region at both 4 M and 12 M, with 12, 27, 83 shared between the 4 M and 12 M cortex, hippocampus, and thalamus, respectively (Fig. 4). Of these proteins, 9 are longitudinally elevated in all three regions, including ANXA3, APOE, APP ARHGDIB, CLU, CST3, CTSS, GFAP, and HTRA1, as displayed in the Venn diagram in Fig. 4b. Also, 2, 8, and 65 longitudinally elevated proteins were unique to the cortex, hippocampus, and thalamus, respectively (Fig. 4b). It is unsurprising that the rTg-DI thalamus displays the greatest number of elevated proteins and degree of overlap, as this region displays the most severe pathology in both emergent and late disease stages.

**Figure 5:**
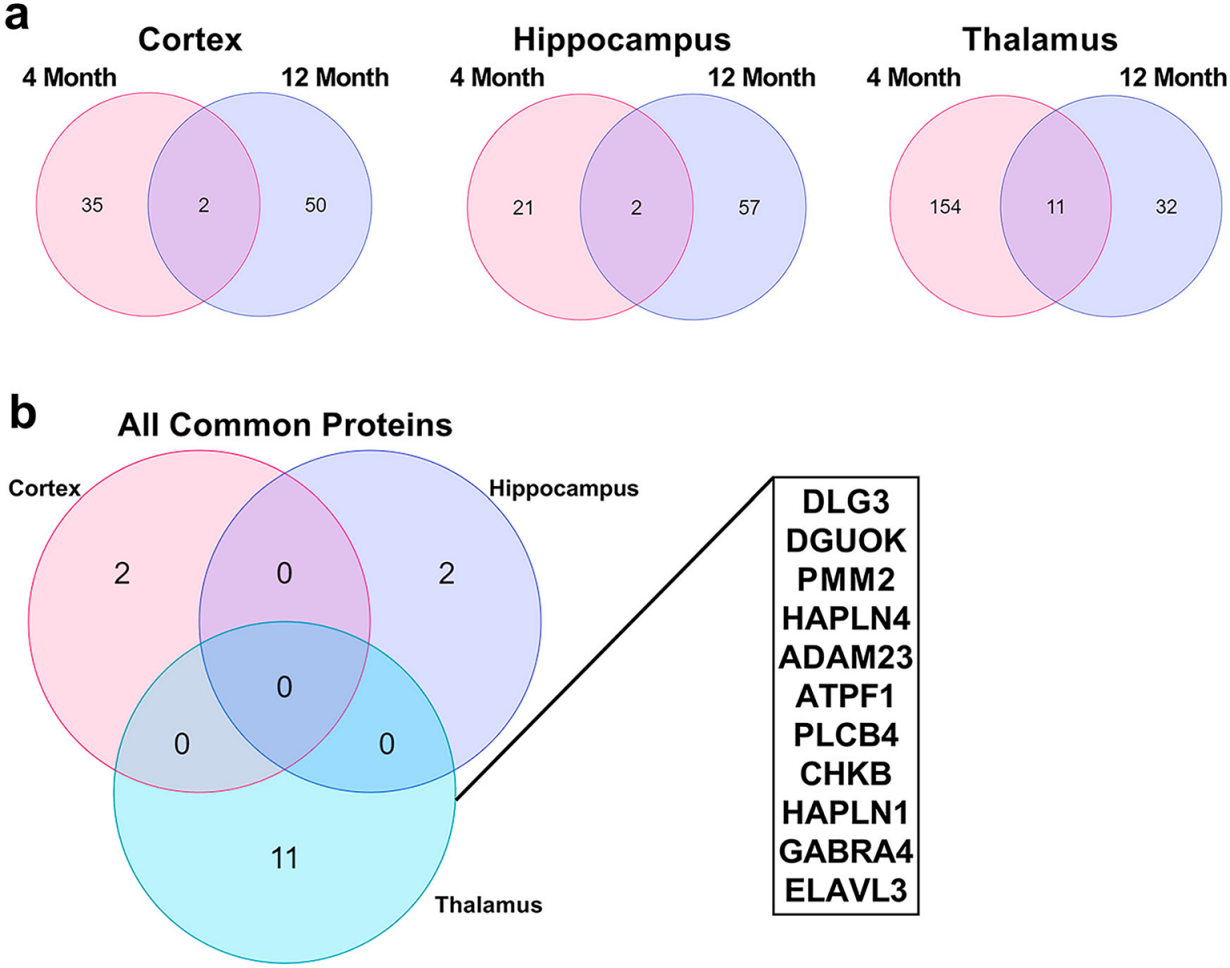
Downregulated protein comparison in 4 M and 12 M rTg-DI brains. (**a**) Venn diagrams comparing significantly decreased proteins (≥ 33% decrease, *p* ≤ 0.05, n = 6) occurring at both 4 M and 12 M in the rTg-DI cortex, hippocampus, and thalamus. (**b**) Venn diagram comparing proteins decreased at both 4 M and 12 M in the cortex, hippocampus, and thalamus, including a list of the 11 proteins commonly reduced in the thalamus at both 4 M and 12 M.

Significantly reduced proteins displayed far less commonality between age groups, as only 11 proteins overlapped in the thalamus (Fig. 5a), and 2 in the cortex or hippocampus, with no common longitudinally reduced proteins among the regions (Fig. 5b). This is not entirely surprising, as many of the proteins we reported with reduced expression in the 12 M cortex and hippocampus are linked to neurodegeneration and WM damage ^20,21^, pathologies not yet observed in the emergent-stage 4 M animals ^18,21^. However, the proteins longitudinally reduced in the thalamus may provide mechanistic insight to the distinctive pathologies emerging there, and are listed in Fig. 5b. Of note among these proteins is hyaluronan and proteoglycan binding link protein (HAPLN4), which is a critical component of perineuronal nets (PNNs), a brain extracellular matrix structure with important roles governing plasticity and modulation of memory^26,27^. Thus, loss of HAPLN4 could suggest a disruption in PNN function in rTg-DI rats potentially contributing to the observed cognitive deficits.

### Longitudinal expression of select DEPs

**Figure 6:**
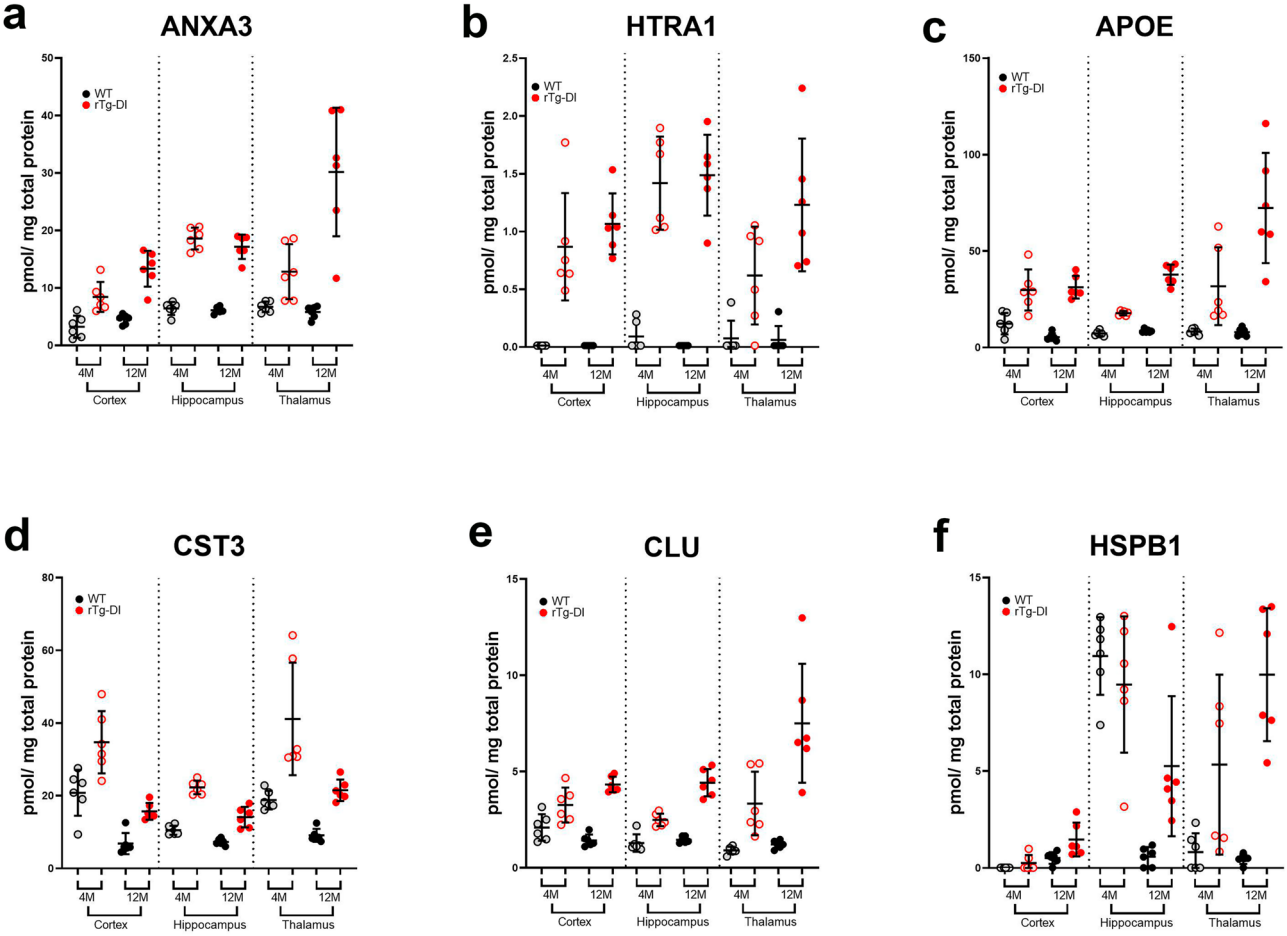
Longitudinal expression of select proteins from 4 and 12 M rTg-DI rat brain regions. Histograms depicting the 4 M and 12 M expression (pmol/mg total protein) of ANXA3 (**a**), HTRA1 (**b**), APOE (**c**), CST3 (**d**), CLU (**e**) and HSPB1 (**f**) in the cortex, hippocampus, and thalamus of WT and rTg-DI rats. Individual points represent individual animals with 4 M WT and rTg-DI shaded with grey and light red, respectively, and 12 M WT and rTg-DI shaded with black and dark red, respectively. Bars represent mean protein concentration in each group (n = 6 ± SD).

We then compared the longitudinal expression of 5 proteins commonly elevated in each region, and 1 protein (HSPB1) elevated in the hippocampus and thalamus, at both emergent and late disease stages. Histograms in Fig. 6a-f depict total protein concentrations for ANXA3, HTRA1, APOE, CST3, CLU and HSPB1 in each region and at both stages of disease with the corresponding WT expression. Apart from HSPB1 in the 4 M cortex, each protein was significantly elevated over the WT expression in each region in both 4 M and 12 M rTg-DI rats. Except for CST3 and HSPB1, enhancement over the WT expression at 4 M increases at 12 M in each region. Early disease enhancement of these proteins with longitudinal increases correlating with disease progression suggests that they may be important markers of CAA and CAA-ri as the disease advances.

### Validation of proteomic analysis and regional marker distribution

**Figure 7:**
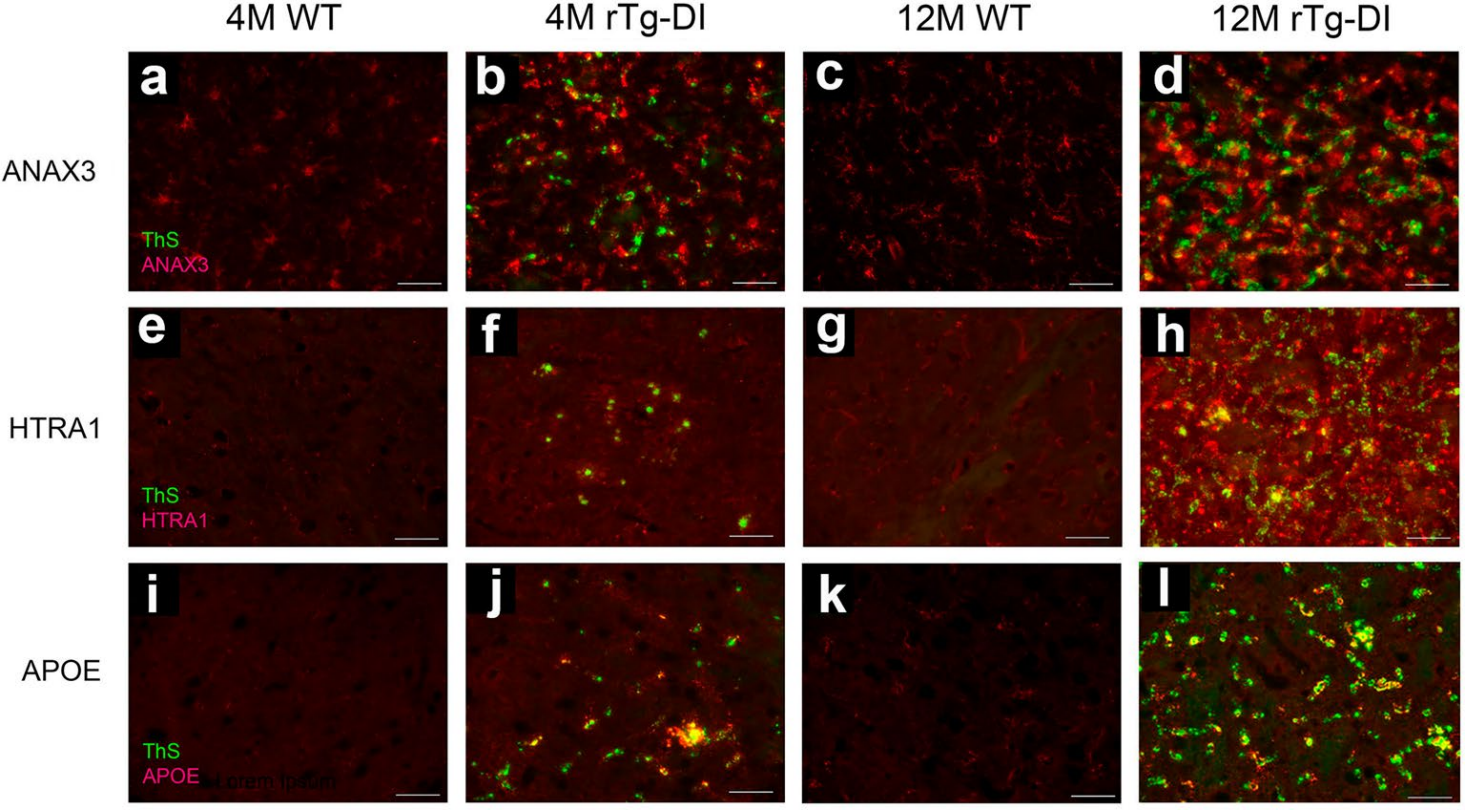
Increased immunolabeling for specific protein markers in rTg-DI rats. Brain sections from 4 M WT rats (**a**,**e**,**i**), 4 M rTg-DI rats (**b**,**f**,**j**) 12 M WT rats (**c**,**g**,**k**) and 12 M rTg-DI rats (**d**,**h**,**l**) were stained with thioflavin S to detect microvascular fibrillar amyloid (green) and rabbit polyclonal antibody to ANXA3 (**a**–**d**), HTRA1 (**e**–**h**) and APOE (i-l) (red). Scale bars = 50 µm. Representative images depicting the thalamic region show each protein is increased in the 4 M and 12 M rTg-DI rats.

We previously identified ANXA3 as specifically elevated in 12 M rTg-DI rats compared with WT rats and the CSVD spontaneously hypertensive stroke prone (SHR-SP) rat model^20–22^. ANXA3 may be an important marker of microglia activation^28^. Despite lower vascular amyloid amounts in initial CAA stages at 4 M, immunolabeling revealed significantly enhanced perivascular ANXA3 signal in the 4 M and 12 M rTg-DI rats surrounding microvascular amyloid deposits (Fig. 7a-d). Furthermore, we have validated upregulation of ANXA3 via Western Blot in pulverized whole brain tissue from 4 and 12 M rTg-DI rats (Figure S2). We have similarly identified HTRA1 as uniquely elevated in 12 M rTg-DI rat brains compared with WT and SHR-SP rats^20–22^. *Htra1* mutations resulting in HTRA1 dysfunction cause the CSVD cerebral autosomal recessive arteriopathy and subcortical infarcts and leukoencephalopathy (CARASIL), and elevation of HTRA1 in human CAA cases has been suggested as a possible marker of CAA^29,30^. Here, immunolabeling confirmed HTRA1 elevated expression in 4 M and 12 M rTg-DI rat brains compared to similarly aged WT rats (Fig. 7e-h). Additionally, we previously showed that APOE is uniquely elevated in 12 M rTg-DI rat brains and co-localizes with microvascular amyloid deposits^20,22^. Here, elevation of APOE in rTg-DI brains at 4 M and 12 M was similarly confirmed along with its co-localization with microvascular amyloid deposits (Fig. 7j,l).

### Pathway analysis indicates activation of TGF-β1

**Figure 8:**
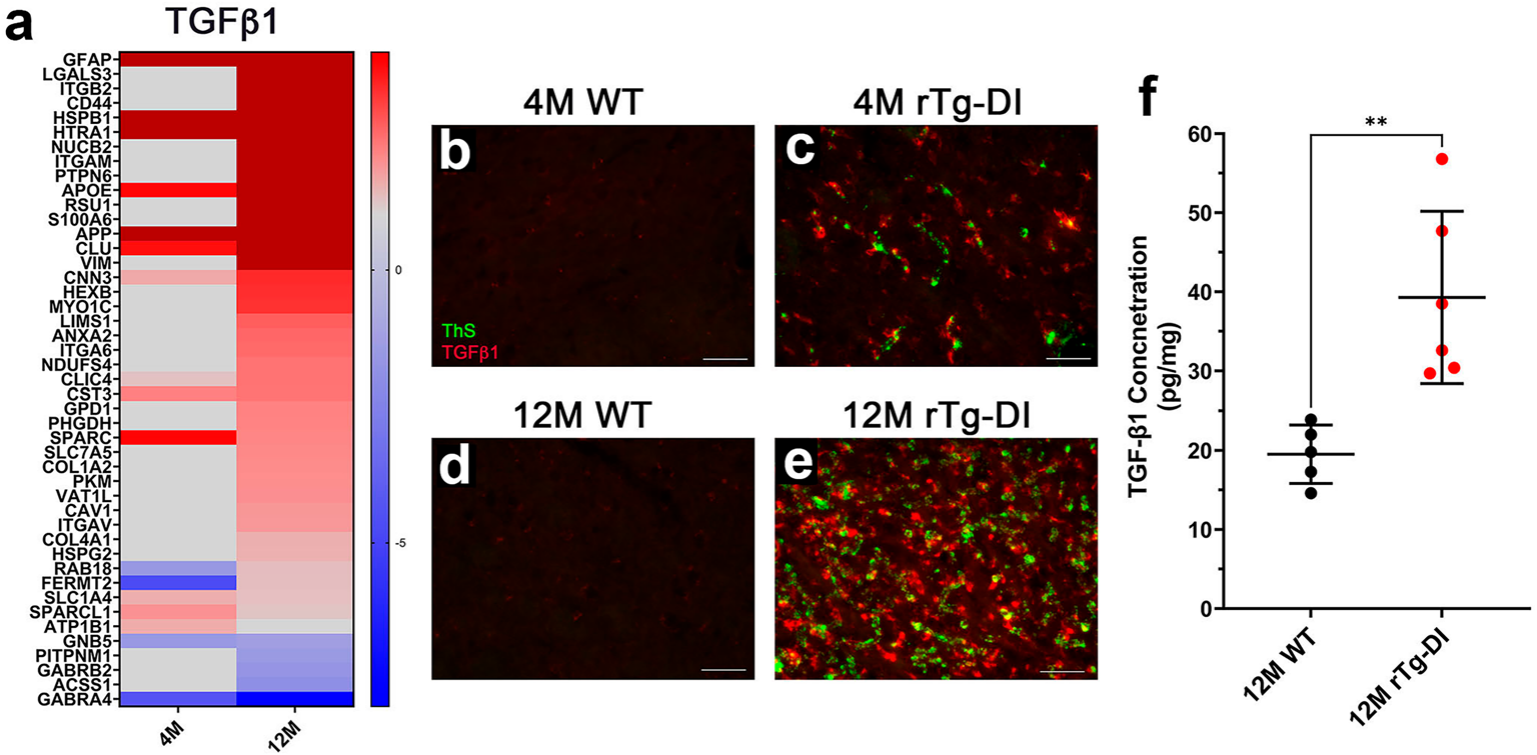
Ingenuity Pathway Analysis (IPA) predicted activation of and validated upregulation TGF-β1 in the rTg-DI thalamus. (**a**) Heat map depicting the differentially expressed proteins (≥ 50% increase or ≥ 33% decrease) in the 4 M and 12 M rTg-DI thalamus associated with the upstream regulator TGF-β1. Red indicates increased, blue indicates decreased, grey indicates not significantly altered expression; color intensity correlates with degree of change as indicated by the fold change reference legend. Brain sections from 4 M WT (**b**), 4 M rTg-DI (**c**) 12 M WT (**d**) and 12 M rTg-DI (**e**) were stained with thioflavin S to detect microvascular fibrillar amyloid (green) and rabbit polyclonal antibody to TGF-β1 (red). Scale bars = 50 µm. Representative images depicting the thalamic region show TGF-β1 is increased in the 4 M and 12 M rTg-DI rats. (**f**) TGF-β1 protein expression (pg/mg) in 12 M rTg-DI and WT rat whole brain tissue as measured by ELISA. Bars represent mean ± SD, and **Indicates p < 0.01. n = 5,6 in the WT and rTg-DI respectively.

To better understand the mechanistic relevance of the overlapping and unique DEPs in each of the age groups, we conducted comparative pathway analysis of the DEPs in the thalamic regions using Ingenuity Pathway Analysis (IPA, Qiagen, Redwood City, CA). IPA analysis was restricted to the thalamic regions as they shared the most overlapping proteins and display the most severe pathological characteristics. IPA predicts activation or inhibition of regulators, networks, and disease functions based on directional expression changes of target proteins, with significance expressed in z scores^31^. Similar to our previous study, IPA analysis of the proteomics data generated here indicated activation of TGF-β1 in the 12 M rTg-DI thalamus (z = 2.212). A heat map comparing the relative expression of DEPs associated with TGF-β1 in the 12 M thalamus and corresponding expression in the 4 M thalamus of rTg-DI rats is displayed in Fig. 8a. Though not meeting the threshold for predicted activation at 4 M, TGF-β1 associated proteins such as HSPB1, GFAP, HTRA1, APOE, CST3, SPARC, and CLU were all elevated. TGF-β1 is reported as a regulator of BBB permeability and integrity, and can harbor both neuroprotective and neurodegenerative functions^32–34^. It is possible that elevation of these proteins during emergent disease stages could indicate initial activation of TGF-β1 and early contributions to the progressive pathologies. Since we did not detect TGF-β1 in our MS analysis, we sought to validate this result by immunolabeling brain tissue. Figure 8b–e confirms a clear increase in TGF-β1 in both the 4 M and 12 M rTg-DI thalamus compared with WT expression. Furthermore, analysis of whole brain tissue by ELISA revealed significant upregulation of TGF-β1 in brains of 12 M rTg-DI rats compared with WT (Fig. 8f). Thus, combining IPA analysis of our proteomic results and validation by immunolabeling and ELISA, we identified TGF-β1 as another protein elevated early in rTg-DI rat brain.

### Differential expression of disease associated astrocytic and microglial proteins in CAA-ri

**Figure 9:**
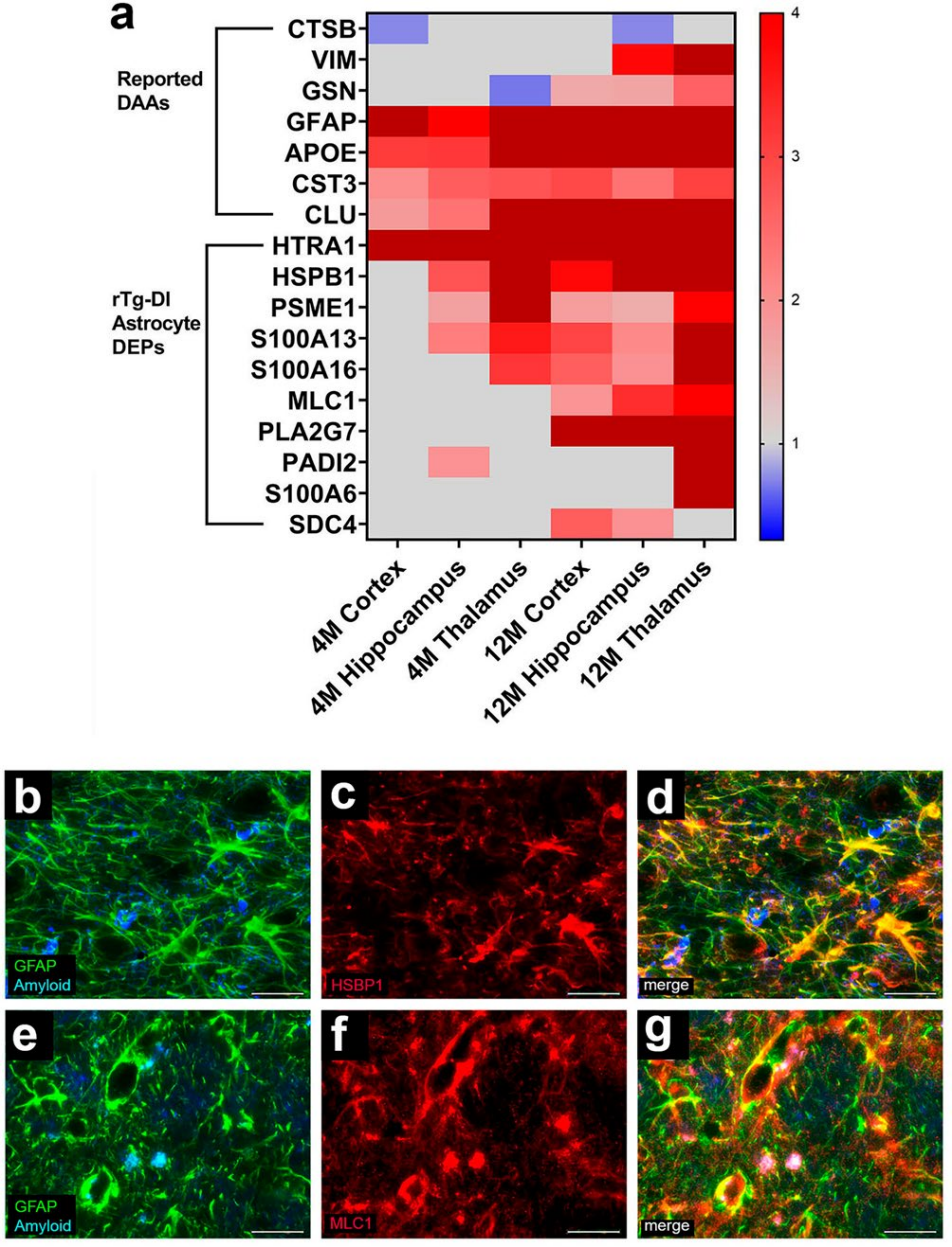
Altered Disease Associated Astrocyte (DAA) and other astrocytic proteins in rTg-DI rats. (**a**) Heat map depicting the relative expression of reported DAA and other astrocytic proteins (≥ 50% increase or ≥ 33% decrease) in the 4 M and 12 M rTg-DI brain regions. Red indicates increased, blue indicates decreased, and grey indicates not significantly altered expression; color intensity correlates with degree of change as indicated by the expression ratio reference legend. Brain sections from 12 M rTg-DI rats were stained with Amylo-glo to detect vascular amyloid deposits (blue) and labeled with rabbit polyclonal antibody to GFAP to detect astrocytes (green) (**b**,**e**) and mouse monoclonal antibodies to detect HSPB1 (**c**) or MLC1 (**f**) (red). Merge images are shown in panels d and g. Scale bars = 50 µm.

Recent studies have reported disease specific subtypes of astrocytes and microglia in models of ADRD revealed by single cell RNA sequencing analysis (scRNA-seq)^35–39^ known as Disease Associated Astrocytes (DAAs) or Disease Associated Microglia (DAMs). Characterization of DAAs and DAMs phenotypes and functions is important, as they likely mediate many of the neuroinflammatory responses specific to amyloid accumulation in ADRD^35,38,40^. Interestingly, many reported upregulated DAA and DAM genes^35–40^ are DEPs we identified in rTg-DI brain regions. A heat map depicting the relative expression of previously reported upregulated DAA proteins is displayed in Fig. 9a, along with other DEPs known to be expressed by astrocytes, that may be novel CAA-ri specific DAAs. As mentioned above GFAP, APOE, CST3, and CLU are elevated in each region in both age groups but are also all reported DAA genes^35^. Other reported DAA genes GSN and VIM were elevated in 12 M brain regions whereas CTSB was reduced in the 4 M cortex and 12 M hippocampus (Fig. 9a). Many astrocytic proteins not previously reported as DAAs were also elevated in multiple regions in both age groups of rTg-DI rats including, HTRA1, HSPB1, PSME1, S100A13, and S100A16, with MLC1 and PLA2G7 which are elevated in all three regions at 12 M (Fig. 9a). This suggests the collective differential expression of these proteins represents a unique astrocytic phenotype in response to CAA-ri occurring in the rTg-DI rat. We validated the astrocytic expression of two of these potential CAA-ri DAAs, HSPB1 and MLC1, via immunolabeling and co-localization with the astrocytic marker and reported DAA gene GFAP depicted in Fig. 9b–g. Both HSPB1 and MLC1 display strong co-localization with GFAP, confirming their upregulation specifically in rTg-DI activated astrocytes, and further highlighting their potential as CAA-ri specific DAAs.

**Figure 10:**
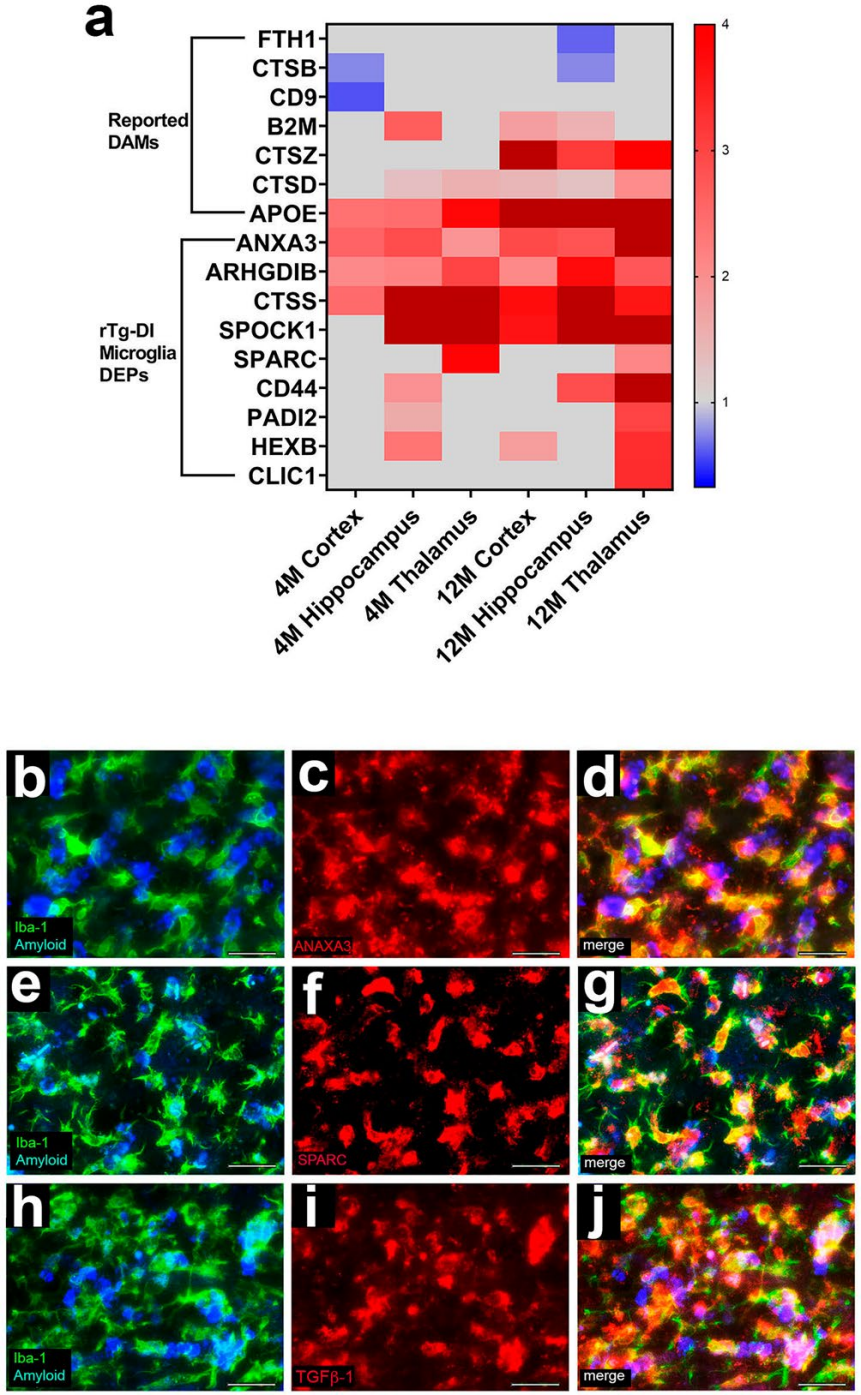
Altered Disease Associated Microglia (DAM) and other microglial proteins in rTg-DI rats. (**a**) Heat map depicting the relative expression of reported DAM and other microglial proteins (≥ 50% increase or ≥ 33% decrease) in the 4 M and 12 M rTg-DI brain regions. Red indicates increased, blue indicates decreased, and grey indicates not significantly altered expression; color intensity correlates with degree of change as indicated by the expression ratio reference legend. Brain sections from 12 M rTg-DI rats were stained with Amylo-glo to detect vascular amyloid deposits (blue) and labeled with rabbit polyclonal antibody to Iba-1 to detect microglia (green) (**b**,**e**,**h**), and mouse monoclonal antibodies to detect ANXA3 (**c**), SPARC (**f**), or TGF-β1 (**i**) (red). Merge images are shown in panels (**d**,**g**,**j**). Scale bars = 50 µm.

Similarly, several previously reported upregulated DAM proteins were detected in our proteomic analysis, though only B2M, CTSD and APOE were elevated in multiple regions in both age groups of rTg-DI rats (Fig. 10a). However, other reported DAM proteins such as FTH1, CTSB or CD9 were either unchanged or down regulated in one or more regions (Fig. 10a). In addition to these proteins, many microglial proteins not previously reported as DAMs were upregulated in the rTg-DI model (Fig. 10a). ANXA3, ARHGDIB, CTSS, SPOCK1, and CD44 were all upregulated in multiple regions in both age groups, with SPARC also upregulated in both the 4 M and 12 M thalamus (Fig. 10a). As only a few of the previously reported DAM proteins are altered in the rTg-DI model, and many other microglial proteins are elevated, this suggests that microglia can adapt a CAA-ri specific DAM phenotype, highlighted by the observed microglia DEP signature. To validate the microglial specific expression of some of these proteins we performed co-immunolabeling of ANXA3, and SPARC along with the microglial marker IBA-1 (Fig. 10b–g). Both ANXA3 and SPARC displayed strong co-localization with IBA-1, indicating their expression specifically in microglia. Based on its specific upregulation in the rTg-DI thalamus at both 4 M and 12 M (see Fig. 8), we also sought to determine the cellular expression of TGF-β1. TGF-β1 expression strongly co-localized with IBA-1, indicating that its enhanced expression occurred in microglia. Thus, TGF-β1 is another potential CAA-ri specific DAM in the rTg-DI rats.

## Discussion

Underlying mechanisms of CAA pathological progression remain poorly understood, and there currently exists no available effective treatment for CAA. Although characteristic pathological findings are evident via neuroimaging in later disease stages (e.g. ICH, microhemorrhages, cortical superficial siderosis), there are currently no validated early stage biomarkers for CAA^3^. An overarching goal of this study was to identify longitudinal DEPs, spanning emergent and late disease stages, that could potentially serve as early biomarkers of CAA, particularly in presence of related inflammation. Validated biomarkers in early disease stages, before the occurrence of hemorrhages and cerebral infarction are crucial for useful diagnosis prior to these advanced disease complications.

Protein mass spectrometry is advantageously suited to deliver precise diagnostic data, and is poised for the identification of potential biomarkers^12,13^. Recently, protein MS analysis of human brain tissue has been utilized for CAA biomarker discovery attempts in human CAA cases^29,30,41,42^. Certainly, a limitation of our study is the potential for species differences between the rTg-DI rat CAA model and human CAA cases. Nevertheless, our proteomic analysis has certain advantages relative to the human based approaches. Firstly, human CAA cases exist on a background of potentially confounding variables, such as genetic, lifestyle, environmental, and medication differences that challenge the identification of CAA-specific proteome alterations. Proteomic analysis of preclinical rodent model populations, such as rTg-DI, are more uniform due to the maintenance of genetic background and consistent environmental conditions. Secondly, proteomic studies of brain tissue from human cases are conducted post-mortem, and thus the analysis represents only advanced CAA disease states. Alternative approaches to CAA biomarker discovery in human biological fluids are being conducted, but case selection is typically dependent on neuroimaging markers of advanced pathologies and, therefore, do not represent emergent disease stages^3,43–45^. Thus, the ability to perform proteomic analysis of the rTg-DI rat model in defined emergent stages of CAA, is a distinct advantage of the present study.

Several of the rTg-DI brain DEPs identified here have potential as biomarkers of CAA, particularly for CAA-ri. ANXA3 was significantly enhanced in each brain region at 4 M, and further increased at 12 M (Figs. 4a,b and 6a). ANXA3 has been previously suggested as a marker of microglia activation ^28,46^, consistent with its localization here (Fig. 10b–d). We reported specific upregulation of ANXA3 in the cortex, hippocampus, thalamus, and corpus callosum, of 12 M rTg-DI^20,21^, and this upregulation remains unique to the rTg-DI CAA model when compared with the CSVD SHR-SP rat model of hypertension^22,47^. Therefore, the longitudinal upregulation of ANXA3 could be an important early marker of CAA-ri progression, and ANXA3 is potentially a CAA-ri specific DAM protein. Also of substantial interest is longitudinal upregulation of HTRA1 (Figs. 6b, 7e–h). HTRA1 is implicated in another CSVD known as CARASIL, where mutations in the *Htra1* gene cause dysfunctional HTRA1, which leads to cerebral small vessel pathology, WM damage and VCID^48–50^. We previously reported upregulation of HTRA1 in the rTg-DI cortex, hippocampus, thalamus, and corpus callosum at 12 M, which also remained unique when compared with the SHR-SP rat model^20–22,47^. Furthermore, consistent with our findings in the rTg-DI rat model, HTRA1 has recently been identified as upregulated and a possible CAA marker in human CAA type-1 cases^29,30^. Thus, HTRA1 may be another important early marker of CAA type-1.

APOE was also elevated in the rTg-DI rats in emergent CAA stages and increased longitudinally with the progression of disease (Figs. 4b and 6c), while immunolabeling, consistent with our previous findings, revealed co-localization with vascular amyloid deposits (Fig. 7i-l). The connection between APOE and AD and the accumulation of Aβ is well documented^51–53^. Furthermore, *Apoe* alleles have been linked to the presence and severity of CAA vasculopathies^54^. Similarly, CST3 has been reported to co-localize with parenchymal and vascular amyloid deposits in the brain, and elevation of CST3 has been reported in the serum and brains of AD patients^55–57^. GFAP is a well-known marker of astrocyte activation/astrogliosis^58,59^, and therefore its longitudinal upregulation in the rTg-DI rat brain is likely not CAA specific. Nevertheless, serum levels of GFAP are being investigated as a biomarker for brain and spinal cord disorders, and proposed as a differentiating tool between AD and other dementias^60,61^. CLU has been linked to AD as a genetic risk factor^62,63^, and in the APP/presenilin 1 (PS1) mouse model, loss of CLU resulted in a marked reduction in parenchymal amyloid deposition but a dramatic increase in CAA^64^. Thus, despite lacking specificity for CAA the proteins APOE, CST3, GFAP, and CLU could be used as a part of a panel of biomarkers to differentiate CAA from non-amyloidal CSVD and potentially other forms of dementia disorders.

The IPA analysis predicted activation of TGF-β1 prompted us to investigate TGF-β1 expression in the 4 M and 12 M thalamus, despite not specifically detecting it in our MS analysis. Immunolabeling confirmed not only robust expression of TGF-β1 in both age groups (Fig. 8b–e) but that TGF-β1 was specifically elevated in microglia (Fig. 10h–j) suggesting it is a CAA-ri specific DAM protein. TGF-β1 is known to induce endothelial to mesenchymal transition which can lead to an increase in BBB dysfunction^33,65^, but is also reported to promote BBB integrity and a tight barrier phenotype in brain endothelial cells^32,66^. Thus, the exact role of TGF-β1 in CAA related pathology is unclear and may have varying effects on BBB integrity. Aberrant regulation of TGF-β1, as a result of dysfunctional HTRA1, has been suggested as a mechanism for the development of CARASIL^50,67^. Thus, the upregulation of both proteins in emergent disease states could be early indication of emerging altered TGF-β1 signaling related to the pathological progression of CAA.

Amyloid pathology specific DAA and DAM phenotypes may harbor critical roles governing neuroinflammatory responses in ADRD, and thus the longitudinal differential expression of multiple DAA and DAM proteins in the rTg-DI brain regions is of particular interest. Numerous studies have reported upregulation of triggering receptor expressed on myeloid cells 2 (TREM2) in microglia as a key indication of a DAM phenotype^36–38,40^. Although we did not detect TREM2 in our proteomics analysis described here, we have previously reported longitudinal upregulation of *Trem2* gene expression in rTg-DI rats beginning at 3 M^17^. APOE has been reported as a DAM gene^36–38^, although APOE is also commonly synthesized and secreted by astrocytes^68,69^, and, therefore, we also included it in our list of astrocytic proteins (Fig. 9a). Though it has not been described as a DAA gene, overlap between DAM and DAAs has been reported^35^, and APOE could be upregulated in DAAs. Therefore, APOE arising from both astrocytic and microglial origins could have mechanistic impacts on CAA progression. Upregulation of many additional non-DAA/DAM astrocytic/microglial proteins in rTg-DI rat brains, many as early as 4 M, highlights glial cell activation in emergent stages and throughout progression of CAA-ri pathologies in the rTg-DI rats, and could point to the presence of CAA-ri specific astrocyte and microglial subtypes. We validated astrocytic expression of MLC1 and HSPB1 (Fig. 9). Previously, we reported enhanced expression of MLC1 in astrocytes in the rTg-DI corpus callosum^21^. Missense mutations in the *Mlc1* gene cause the leukodystrophy megalencephalic leukoencephalopathy with subcortical cysts^70^. Diffuse WM loss in the rTg-DI corpus callosum is pronounced^18,21^, and with MLC1 already implicated in leukodystrophy, it is possible that MLC1 upregulation is of mechanistic importance in the astrocytic response as a CAA-ri specific DAA. HSPB1 release from astrocytes in response to Aβ exposure has been demonstrated *in-vitro*^71^, and HSPB1 overexpression enhanced neuronal excitability and restored long-term potentiation in the APP/PS1 mouse model of AD^72^. Furthermore, upregulation of HSPB1 mediated by TGF-β1 has been reported^73^, and HSPB1 has been shown to be protective of BBB integrity in models of cerebral ischemia and stroke^74,75^. It is possible that HSPB1 upregulation in emergent disease stages is stimulated by either Aβ deposition, or TGF-β1 upregulation, and may serve BBB protective functions. Thus, HSPB1 is of great interest both as a potential biomarker and mechanistic importance as a CAA specific DAA. Finally, SPARC was upregulated specifically in microglia (Fig. 10). SPARC has been suggested as a key initiator of cerebral inflammation in AD and BBB permeability as well, thus may be another CAA-ri marker^76^.

### Future Directions

Early, longitudinal, and specific upregulation of proteins identified here is promising for the identification of CAA specific biomarkers and mechanisms contributing to disease progression, particularly CAA-ri. Distinctive differential expression will be a vital component for any potential biomarker, and thus in addition to the SHR-SP model of hypertension comparison already reported^22,47^, the DEPs identified here should be compared with other CSVD models, including models of larger vessel CAA type-2 or a model prominently exhibiting Aβ plaque and tau AD pathologies. Additionally, follow up investigation of DEPs identified here for detection in biological fluids such as cerebrospinal fluid (CSF) and blood plasma will be necessary. DEPs that remain specific to the CAA rTg-DI model and are detectable in biological fluids will harbor the greatest potential as diagnostic biomarkers, and these should then be similarly investigated for differential expression in human CAA cases. The CAA relevant functions of ANXA3, HTRA1, SPARC and TGF-β1 are also of great interest. ANXA3 upregulation in the rTg-DI rat does not simply mimic microglial enhancement^21^, and therefore may have important molecular functions in microglia activated by cerebral vascular amyloid. HTRA1 dysfunction and its contributions to CARASIL have been reported, however impacts of HTRA1 upregulation in the context of CAA is yet to be determined. Therefore, the biological functions of HTRA1 and its impacts on CAA progression need to be further investigated. Further characterizing the potential CAA-ri specific astrocyte and microglia subtypes is an important line of investigation. Characterization of CAA-specific signaling within these cell types may yield novel therapeutic targets and deeper understanding of CAA pathological mechanisms. Future studies employing scRNA-seq or single-cell proteomics to characterize specific astrocyte and microglia populations in the rTg-DI model need to be conducted. Finally, several new passive immunization therapies for AD that target Aβ are available to patients^77,78^. A shared potentially serious complication of these therapies is the development of amyloid related imaging affects (ARIA) including hemorrhages^77,79,80^. CAA is recognized as a strong risk factor for developing immunotherapy induced ARIA^3,80,81^. Therefore, it will be important to identify patients with a high CAA burden where immunotherapy targeting Aβ would be contraindicated. In conclusion, the data presented here have identified potential new biomarkers for CAA, particularly CAA-ri, where further validation and development may be useful for early detection and monitoring progression of this condition.

## Methods

### Animals

All work with animals was approved by the University of Rhode Island Institutional Animal Care and Use Committee and in accordance with the United States Public Health Service’s Policy on Humane Care and Use of Laboratory Animals was in compliance with the ARRIVE guidelines^82^. The rTg-DI rat model, expressing low levels of human Swedish/Dutch/Iowa mutant AβPP under control of neuronal specific Thy1.2 promoter producing chimeric Dutch/Iowa CAA Aβ peptides in the brain, was generated as previously described^16^. 4 M and 12 M rTg-DI rats and Sprague Dawley wild-type (WT) rats (3 females + 3 males per group) were used for this study. All rats were housed in a controlled room (22 ± 2 °C and 40–60% humidity) on a standard 12 h light cycle. Rat chow and water were available ad libitum.

### Brain tissue collection and preparation

Anesthetized rats were transcardially perfused with PBS and the brains surgically removed. Rat brains were then bisected in the mid-sagittal plane, with one hemisphere subsequently fixed in 4% paraformaldehyde (PFA) for immunohistochemical analysis and the other hemisphere placed in OCT and frozen at − 80 °C.

### Laser capture microdissection and protein digest

Laser capture microdissection of cortical, hippocampal, and thalamic regions from 4 M rTg-DI and WT rats was performed as previously described^20^. Protein isolation and digestion was performed as previously described^20,21,47^. Briefly, isolated tissue was lysed in 1× radioimmunoprecipitation assay (RIPA) buffer via sonication and incubation on ice for 2 h, and proteins were denatured by the addition of DTT (20 mM final concentration) and 15 min incubation at 95 °C with shaking. Alkylation was performed by addition of iodoacetamide (IAA) and incubation at room temperature in the dark for 30 min. Proteins were precipitated and concentrated via chloroform methanol precipitation (2:1:1 methanol : water : chloroform) and resuspended in sodium deoxycholate (3% w/v in 50 mM ammonium bicarbonate). Proteins were digested with TPCK-treated trypsin using pressure cycling technology in a barocycler (Pressure Bioscience Inc, Easton, MA) as previously described^20–22,47^.DOC was then precipitated by the addition of formic acid (in 50% v/v acetonitrile and water, 0.5% v/v final concentration), followed by centrifugation, and supernatant collected for analysis by mass spectrometry.

### Analysis by LC-QTOF/MS

All proteomic experiments were performed as previously described^20–22,47^. Briefly, experiments were conducted on a SCIEX 5600 TripleTOF mass spectrometer in positive ion mode utilizing a DuoSpray™ ion source (AB Sciex, Concord, Canada) following chromatographic separation with an Acquity UPLC H-Class system (Waters Corp., Milford, MA) using an Acquity UPLC Peptide BEH C18 (2.1 X 150 mm, 300 Å, 1.7 µm) column preceded by an Acquity VanGuard pre-column (2.1 X 150 mm, 300 Å, 1.7 µm). All mass spec settings were exactly as previously described^20–22,47^. Data was acquired in data independent acquisition mode (DIA) using Analyst TF 1.7.1 software (AB, Sciex).

### Data Processing

Raw data was analyzed with Spectronaut™ (Biognosys, Schlieren, Switzerland) software as previously described^20–22,47^. All settings were kept at Spectronaut factory defaults except “used Biognosys’ iRT kit” and “PTM localization” were deselected, and normalization set to “local”. Proteins were identified and quantified from DIA data according to the Spectronaut™ Pulsar™ algorithm, referencing our previously formed in-house spectral library^20^ combined with the fractionated rat brain, liver, kidney Biognosys Standard Spectral library. A False Discovery Rate of 0.01 was managed by Spectronaut at the protein, peptide, and protein spectrum match levels. Protein intensities from the Spectronaut output were then converted to molar concentrations (pmol / mg total protein) according to the total protein approach (TPA)^83^. As done previously, we imputed a baseline concentration of 0.013 pmol/mg for protein concentrations of zero (filtered by Spectronaut for low intensity) in individual samples. Although TPA concentrations lower than this baseline were calculated here, this value is based on lowest calculated TPA concentrations across multiple Spectronaut analyses. Average molar concentrations were compared to identify differentially expressed proteins in the 4 M and 12 M brain regions of rTg-DI rats. As previously performed^20–22,47^, we chose to consider uncorrected *p* values and to manage the false discovery rate (FDR) by implementing effect threshold cutoffs. Differentially expressed proteins (DEPs) were thus defined as proteins with a ≥ 50% increase or ≥ 33% decrease in expression compared to similarly aged WT animals and a *p* value of ≤ 0.05. DEPs were identified by head-to-head comparison of average protein concentrations between groups, and statistical significance determined by *t* test with p values ≤ 0.05 considered significant.

### Immunolabeling and histological analyses

Frozen PFA-fixed brain hemispheres were cut in the sagittal plane at 20–50 µm thickness using a cryostat (Leica, Buffalo Grove, IL) and tissue sections were placed on slides. Slides were incubated for 5 min with proteinase K (0.2 mg/ml) at 22 °C for antigen retrieval. Tissue section blocking occurred via 30 min incubation with Superblock blocking buffer (37,518, ThermoFisher) containing 0.3% Triton X-100 at room temperature, and primary antibody incubations occurred overnight at the following dilutions: rabbit polyclonal antibody to collagen IV to identify cerebral blood vessels (1:250, SD2365885, Invitrogen); goat polyclonal antibodies to glial fibrillary acidic protein (GFAP, 1:250, ab53554, Abcam) or ionized calcium-binding adapter molecule 1 (Iba-1, 1:250, NB100-1028, Novus) used to identify astrocytes and microglia, respectively, and rabbit polyclonal antibodies to ANXA3 (1:250, PA5082483, Invitrogen), HTRA1 (1:200, MAB2916, R&D Systems), MLC1 (1:200, PS5-41042, Invitrogen), and rabbit monoclonal antibodies to (APOE (1:250, RRID: AB_2832971, Abcam, Cambridge, MA), TGF-β1 (1:200, AB_215715, Abcam), and SPARC (1:200, AB_290639, Abcam) and mouse monoclonal antibody to HSPB1 (1:200, AB_215715, Abcam). Primary antibodies were detected using Alexa Fluorescent 594- or 488-conjugated secondary antibodies (1:1000). Thioflavin S (123H0598, Sigma-Aldrich) or Amylo-Glo (TR-300-AG, Biosensis Inc.) staining were used for the detection of fibrillar vascular amyloid deposits as described by the manufacturer. Immunolabeled and histological images were collected with the Keyence BZ-X710 Microscope (RRID:SCR_017202) and analyzed with the Keyence BZ-X Analyzer Software Version 1.3.1.1 (Keyence Corp. Osaka, Japan).

### Immunoblot Analysis

Whole brain tissue from 4 and 12 M rTg-DI and WT rats were lysed in 1X RIPA buffer containing protease and phosphatase inhibitor cocktails (ThermoFisher Scientific, RID#A32953, and A32957) via sonication (12 × 1 s bursts) and incubated on ice for 1 h, and samples were normalized to equal protein concentrations. Following resolving by SDS-PAGE, proteins were transferred to polyvinylidene difluoride (PVDF) membrane (Imobilon-FL, EMD Millipore, Billerica, MA). Relative expression of ANXA3 was revealed via probing with rabbit polyclonal antibody to ANXA3 (1:250, PA5082483, Invitrogen), and IRDye® 800CW goat anti-Rabbit IgG secondary antibody (LI-COR, RRID# AB_621843). ANXA3 signal was normalized against β-actin using mouse monoclonal anti-β actin primary antibody (Sigma, A5441) and IRDye® 680RD goat anti-mouse secondary antibody (LI-COR, RRID# AB_10956588).

### Enzyme-linked immunosorbent assay

Whole brain tissue from 12 M rTg-DI and WT rats were lysed and normalized as described above. TGF-β1 protein concentration in each sample was revealed using the TGF-β1 (LAP) Rat Uncoated ELISA kit (ThermoFisher Scientific, RRID#88–50680) according to the manufacturer’s guidelines.

### Supplementary Information

Supplementary Information 1.

Supplementary Information 2.

Supplementary Information 3.

Supplementary Information 4.

Supplementary Information 5.

Supplementary Information 6.

Supplementary Information 7.

## Data Availability

The data sets used and/or analyzed during the current study are available from the corresponding author on reasonable request. Raw mass spectrometry data can be found in the MassIVE repository (massive.ucsd.edu/ProteoSAFe/static/massive.jsp), project ID#: MSV000093166, and password: rTgDI4MReg, as well as the project ID#: MSV000086432 and password: rTgDIrgprt1219 for previous 12 M animal raw spectral files previously published^20^.
