# Supplementary material for: Longitudinal markers of cerebral amyloid angiopathy and related inflammation in rTg-DI rats
Source: Sci Rep. 2024 Apr 10;14:8441. doi: 10.1038/s41598-024-59013-7 (PMC11006668; doi:10.1038/s41598-024-59013-7)
Supplement: Supplementary file 1 — Supplementary Information 1. [file 41598_2024_59013_MOESM1_ESM.docx]

**Longitudinal markers of cerebral amyloid angiopathy and related**

**inflammation in rTg-DI rats**

Joseph M. Schrader^1^, Feng Xu^1^, Kevin J. Agostinucci^1^, Nicholas A. DaSilva^2^, and William E. Van Nostrand^1^*

**SUPPLEMENTAL INFORMATION**


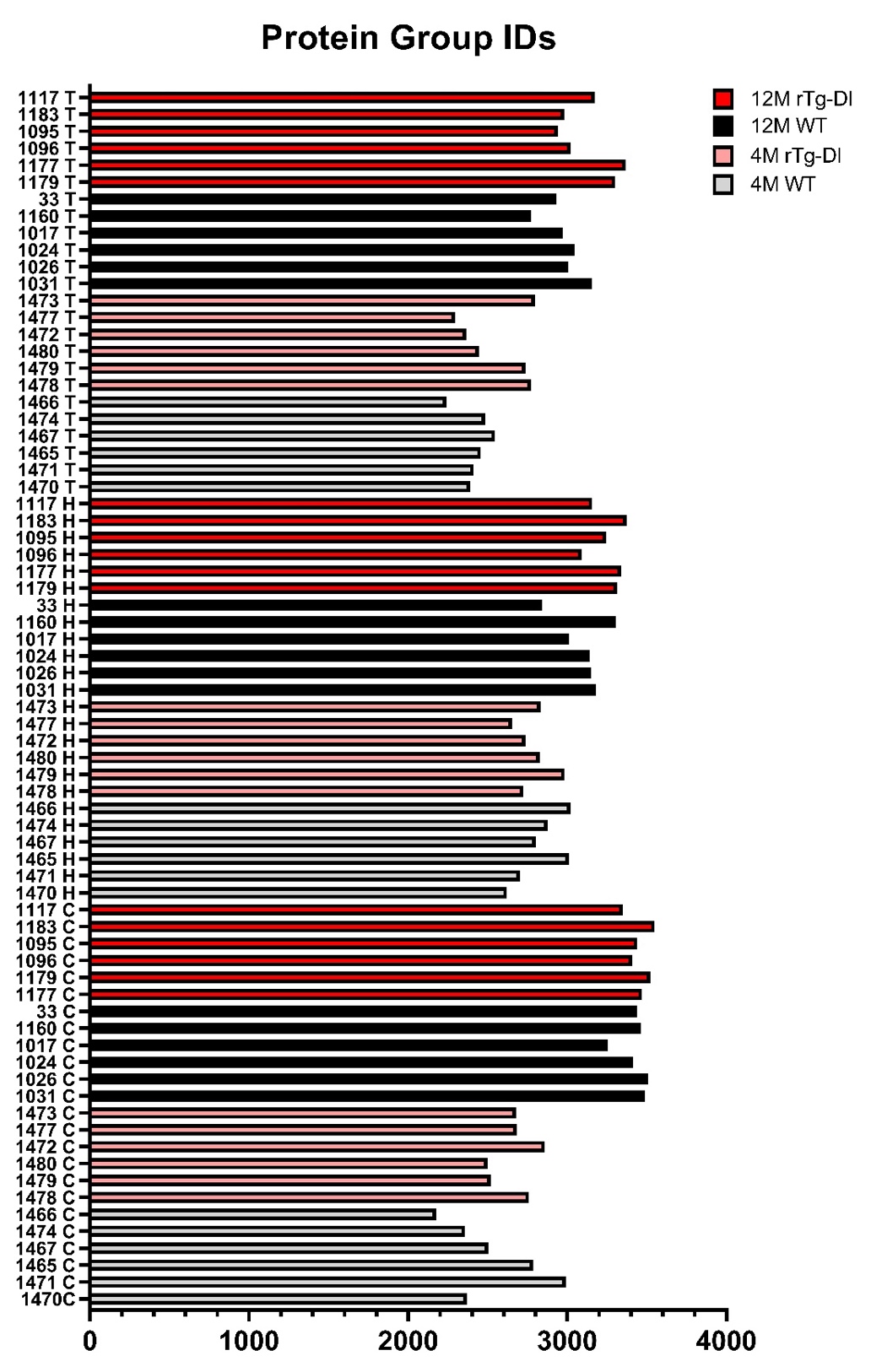


**SI Figure S1. Protein Group Identifications by Sample.** Bar graph displaying the number of unique protein groups identified in each sample. 12M and 4M rTg-DI samples are colored in red and light red, respectively, while 12M and 4M WT samples are colored in black and grey, respectively. Brain regions are indicated as C for cortex, H for hippocampus, and T for thalamus, and individual animal ID numbers are indicated.

**
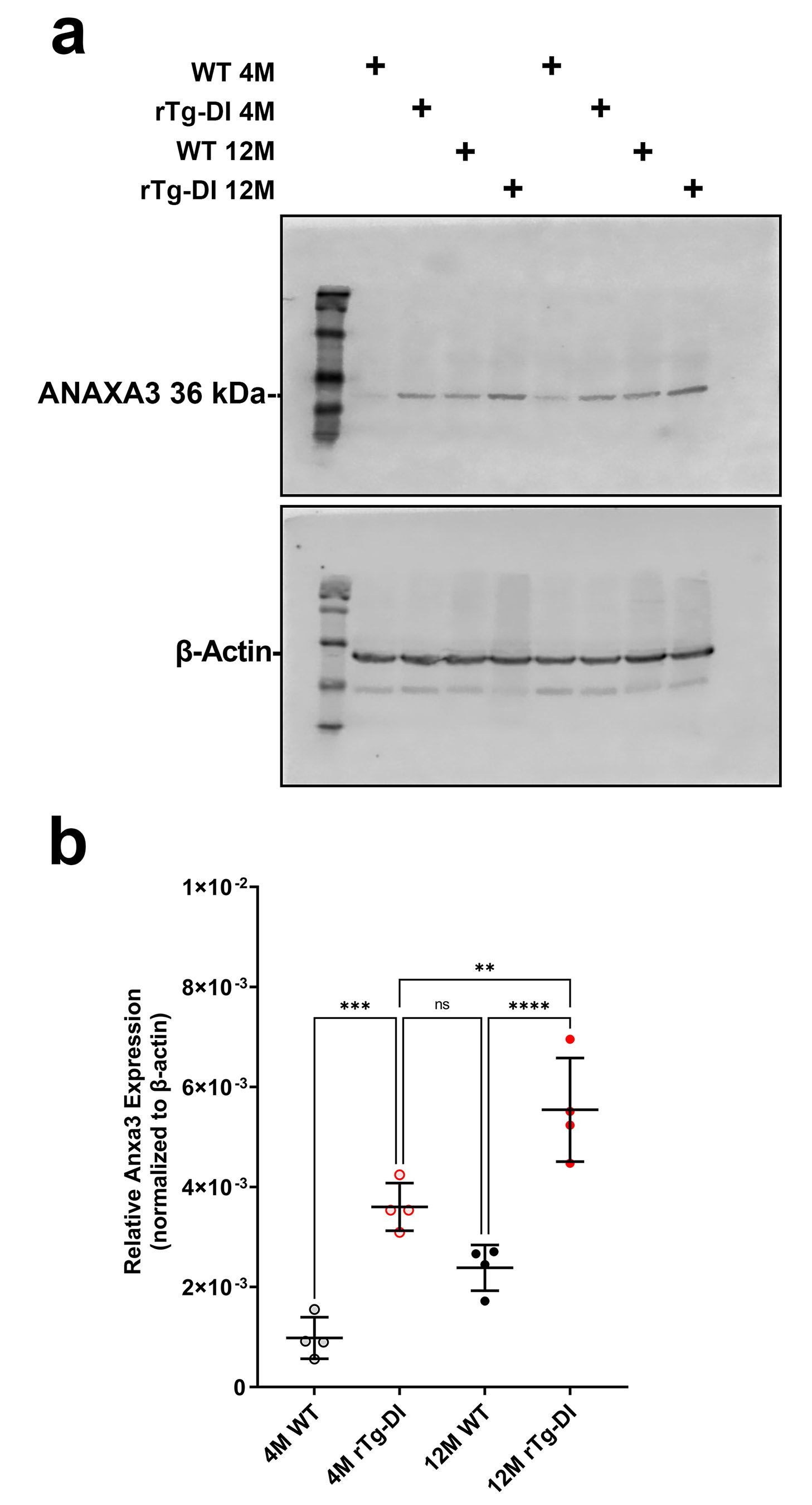
**

**SI Figure S2. Validation of ANAXA3 Upregulation by Immunoblot.** (**A**) Quantification of relative whole brain expression of ANAXA3 in rTg-DI and WT at 4M and 12M normalized to β-actin. Individual values are depicted with bars representing the mean ± SD. n = 4, ***p* = 0.055, ****p* = 0.0005, *****p* < 0.0001, determined by one-way ANOVA. (**B**) Representative immunoblot depicting relative whole brain expression of ANAXA3 in rTg-DI and WT rats and used for quantification in (**A**). Relative ANAXA3 signal was revealed by probing with rabbit polyclonal anti-ANAXA3 primary antibody, and subsequent fluorescent secondary antibody. Relative β-actin signal (bottom) was revealed by probing with mouse monoclonal anti-β-actin primary antibody and subsequent fluorescent secondary antibody and used as a loading control, as β-actin is not changed in rTg-DI rats. Images are from the same immunoblot in alternate fluorescent channels.

**SI Tables:**

**Table S1. 4M rTg-DI Cortex Differentially Expressed Proteins**

**Table S2. 4M rTg-DI Hippocampus Differentially Expressed Proteins**

**Table S3. 4M rTg-DI Thalamus Differentially Expressed Proteins**

**Table S4. 12M rTg-DI Cortex Differentially Expressed Proteins**

**Table S5. 12M rTg-DI Hippocampus Differentially Expressed Proteins**

**Table S6. 12M rTg-DI Thalamus Differentially Expressed Proteins**
